# Distinct prognostic value of aspartate-to-alanine aminotransferase ratio (AAR) in traumatic brain injury versus hemorrhagic stroke: a cohort study of 1,069 patients

**DOI:** 10.3389/fneur.2026.1796615

**Published:** 2026-06-04

**Authors:** Dilmurat Gheyret, Xiaotao Gao, Ziwei Zhou, Lei Li, Jinchao Wang, Xu Zhang, Haoran Jia, Shu Zhang, Mirzat Turhon, Maimaitili Aisha, Cong Wang, Yuhan Li, Yingsheng Wei, Shuo An, Jian Sun, Jianning Zhang, Hengjie Yuan, Ye Tian

**Affiliations:** 1 Department of Neurosurgery, Tianjin Medical University General Hospital, Tianjin, China; 2 Tianjin Neurological Institute, Tianjin, China; 3 Graduate School, Tianjin Medical University, Tianjin, China; 4 School of Medicine, Nankai University, Tianjin, China; 5 Department of Neurosurgery, Beijing TianTan Hospital, Capital Medical University, Beijing, China; 6 Department of Neurosurgery, Xinjiang Medical University Affiliated First Hospital, Urumqi, Xinjiang, China

**Keywords:** AST/ALT ratio, disease heterogeneity, intracerebral hemorrhage, prognostic biomarker, subarachnoid hemorrhage, systemic metabolic response, traumatic brain injury

## Abstract

**Background:**

The liver-brain axis influences outcomes after acute brain injury, yet the prognostic significance of liver markers across varying injury types remains unclear. We examined the disease-specific prognostic value of the aspartate aminotransferase-to-alanine aminotransferase ratio (AAR) in traumatic brain injury (TBI), intracerebral hemorrhage (ICH), and aneurysmal subarachnoid hemorrhage (aSAH).

**Methods:**

This retrospective cohort study included 1,069 consecutive patients (413 TBI, 490 ICH, 166 aSAH). The primary outcome was unfavorable neurological status at discharge (modified Rankin Scale 3–6). Multivariable logistic regression and formal interaction testing assessed the heterogeneity of AAR’s effect across disease types.

**Results:**

Unfavorable outcomes occurred in 420 patients (39.3%). Higher AAR was associated with poor outcomes in the overall cohort (adjusted OR 1.34, 95% CI: 1.07–1.66). However, significant effect modification by disease type was observed (P for interaction = 0.001). In TBI, elevated AAR was a robust independent predictor (adjusted OR 2.15, 95% CI: 1.46–3.15, *p* < 0.001), with the magnitude of association escalating with injury severity (Severe TBI: adjusted OR 5.38). Conversely, AAR showed no significant prognostic value in ICH (*p* = 0.746) or aSAH (*p* = 0.810). A significant dose–response relationship between AAR and unfavorable outcome was observed across the overall cohort (P for trend < 0.001), and the prognostic effect of AAR in TBI escalated markedly with injury severity.

**Conclusion:**

The prognostic value of AAR is highly heterogeneous. It serves as a powerful predictor in TBI—likely reflecting systemic metabolic stress—but lacks utility in hemorrhagic stroke. These findings suggest distinct pathophysiological mechanisms driving liver-brain interactions across neurosurgical conditions, cautioning against a “one-size-fits-all” biomarker approach.

## Introduction

Acute brain injury (ABI) includes a range of sudden-onset neurological conditions, such as traumatic brain injury (TBI), aneurysmal subarachnoid hemorrhage (aSAH), and intracerebral hemorrhage (ICH). Despite their different causes, these conditions share standard pathophysiological mechanisms and clinical challenges. TBI affects 27.08 million people each year, with 8.1 million experiencing disability worldwide (1), while ICH has mortality rates over 40% at 30 days (2). Evidence suggests there may be interactions between brain injury and other body organs (3, 4). One way a brain injury may impact liver function is through the inflammatory response. In animal studies, following acute brain damage, chemokine production in the liver attracts neutrophils, which can lead to liver damage (5). In patients with TBI specifically, liver function test abnormalities occur in approximately 9% of cases, with ALT levels above the normal range commonly observed at admission (6). Hepatic encephalopathy demonstrates how liver dysfunction can impair detoxification, allowing neurotoxins like ammonia and manganese to enter brain circulation and affect brain function (7). As a result, neurosurgical procedures in cirrhotic patients have high complication rates (87.2%) and mortality rates up to 34.5% (8).

The aspartate aminotransferase (AST) to alanine aminotransferase (ALT) ratio (AAR), also known as the De Ritis ratio after its original describer, Fernando De Ritis, has become a valuable prognostic marker across various clinical conditions (9). This ratio offers unique insights beyond individual enzyme levels, as it reflects the different distribution and cellular localization of these transaminases. While ALT is mainly found in hepatocytes, AST exists in both cytoplasmic and mitochondrial compartments across multiple organs, including the brain, heart, muscle, and liver (10). In neurological conditions, the AAR has shown significant prognostic value. A study of 478 acute ischemic stroke patients found that an AAR > 1.53 at admission was linked to a 2.15-fold higher risk of poor 3-month outcomes (95% CI: 1.14–4.05, *p* = 0.018) (9). The prognostic significance likely relates to mitochondrial dysfunction, as elevated ratios suggest a preferential release of mitochondrial AST and impaired glutamate metabolism, which can worsen excitotoxic injury (10–12).

Despite growing awareness of the liver-brain axis, few studies have directly compared the prognostic role of liver biomarkers across different etiologies of acute brain injury within a single cohort. Prior studies have suggested that the AAR may carry prognostic information in traumatic brain injury and stroke, while other readily available systemic markers such as albumin, hyperglycemia, and creatinine have also been explored in neurocritical care settings (13–15). However, most of these investigations were conducted within single-disease populations, limiting our understanding of whether such biomarkers reflect shared neurocritical illness biology or disease-specific pathophysiological signatures. We hypothesized that these distinct pathophysiological profiles would result in differential prognostic values for AAR. Specifically, we postulate that AAR acts as a marker of systemic stress and mitochondrial dysfunction in TBI, distinguishing it from hemorrhagic strokes. To test this, we analyzed 1,069 patients to determine whether the AAR–outcome relationship is modified by the type of brain injury.

## Materials and methods

### Study design and setting

This retrospective cohort study was conducted at the Department of Neurosurgery, Tianjin Medical University General Hospital, China. The institutional review board approved the study protocol, and the need for informed consent was waived due to the retrospective nature of the study.

### Study population

We included all consecutive patients aged 18 years or older admitted to the neurosurgery department between January 2018 and December 2020 with specific diagnoses of TBI, ICH, or aneurysmal subarachnoid hemorrhage (aSAH). Inclusion criteria were: (1) a primary diagnosis of TBI, ICH, or aSAH requiring hospitalization; (2) availability of liver function tests during admission; and (3) documented functional outcome at discharge using the modified Rankin Scale. Exclusion criteria included: (1) pre-existing severe liver disease such as cirrhosis, hepatitis B or C infection, or autoimmune hepatitis; (2) use of hepatotoxic medications before admission; (3) alcohol use disorder; (4) malignancy with known hepatic involvement; (5) incomplete medical records; and (6) discharge within 24 h of admission.

### Data collection and variables

#### Clinical and demographic variables

Patient demographics, medical history, and clinical characteristics were obtained from electronic medical records. Variables included age, sex, primary neurosurgical diagnosis, surgical intervention status, ICU admission, length of ICU stay, Glasgow Coma Scale (GCS) score at admission, and onset-to-admission time. Onset-to-admission time was defined as the interval in hours from injury onset to hospital admission for patients with TBI, and from symptom onset to hospital admission for patients with ICH or aSAH. For patients with multiple concurrent diagnoses, the primary diagnosis requiring hospitalization was used for classification. Traumatic subarachnoid hemorrhage was classified under TBI rather than aSAH. We also systematically recorded medications administered during hospitalization that could potentially affect liver function, including antibiotics, anticonvulsants, hemostatic agents, acid suppressants, hepatoprotective agents, and albumin supplementation.

#### Liver function assessment

All liver function tests performed during hospitalization were systematically collected and analyzed. For each liver function parameter, we determined the minimum, maximum, mean, median, first measurement, last measurement, absolute change, percentage change, and total number of measurements. The primary exposure variable was AAR, calculated as the ratio of AST to ALT using median AST and ALT values during hospitalization. This summary measure was selected to reduce the influence of isolated extreme values and to better reflect the overall in-hospital transaminase profile. To address potential concerns regarding biomarker timing, we additionally calculated the first available AAR using the first recorded AST and ALT values after admission and performed sensitivity analyses using this first-measured AAR.

#### Acute liver injury classification

Patients were classified based on established criteria for liver injury severity and type (16). ALT elevations determined severity levels: (1) Mild injury: ALT 2–5 times the upper limit of normal (ULN), (2) Moderate injury: ALT 5–15 times ULN, and (3) Severe injury: ALT ≥10 times ULN with INR ≥ 2.0 and total bilirubin ≥3.0 mg/dL. Injury pattern classification used the R ratio, calculated as R = (ALT/ULN) divided by (ALP/ULN). Patients were categorized as: (1) Hepatocellular injury (R ≥ 5), (2) Mixed injury (2 < R < 5), or (3) Cholestatic injury (R ≤ 2). These classifications were based on peak laboratory values during hospitalization, with standard reference ranges defined as ALT ≤ 40 U/L and ALP ≤ 120 U/L.

### Outcome measures

The primary outcome was functional status at hospital discharge, measured with the modified Rankin Scale (mRS). Outcomes were divided into favorable (mRS 0–2) and unfavorable (mRS 3–6). Secondary outcomes included in-hospital mortality and functional status at 6 months and 1 year follow-up.

### Statistical analysis

Statistical analyses were conducted using R software (R version 4.3.2, R Foundation for Statistical Computing, Vienna, Austria). Continuous variables were tested for normality with the Shapiro–Wilk test, presented as medians with interquartile ranges (IQRs), and compared using the Mann–Whitney U test or Kruskal–Wallis test, as appropriate. Categorical variables were shown as frequencies with percentages and compared with the chi-square or Fisher’s exact test.

Multivariable logistic regression was used to identify factors associated with unfavorable neurological outcome. Candidate variables included demographic factors, disease type, clinical severity, ICU-related variables, laboratory parameters, and onset-to-admission time. AAR was included based on biological plausibility and the primary study hypothesis. Multicollinearity was assessed using variance inflation factors (VIF < 5). Model discrimination was evaluated using the area under the receiver operating characteristic curve.

To assess whether the association between AAR and outcome differed by disease type, we performed interaction analyses by including a Disease Type × AAR interaction term in the multivariable model. Heterogeneity of effects was evaluated using the likelihood ratio test. Sensitivity analyses were performed by additionally adjusting for onset-to-admission time and by repeating the analysis using the first available AAR measurement instead of the median in-hospital AAR.

## Results

### Study population and baseline characteristics

Between January 2018 and December 2020, a total of 1,069 consecutive patients met the inclusion criteria. The overall cohort included 413 (38.63%) patients with TBI, 490 (45.84%) with ICH, and 166 (15.53%) with aSAH. Onset-to-admission time was available for all patients. The median onset-to-admission interval was 5 h overall, with disease-specific medians of 5 h in TBI, 4 h in ICH, and 4 h in aSAH. These data indicate that most patients presented during the early acute phase of injury or symptom onset (Table 1).

**Table 1:** Baseline characteristics of the study population.

Variables	Total (*n* = 1,069)	Favorable outcome (*n* = 649)	Unfavorable outcome (*n* = 420)	*p*
Male sex, *N* (%)	681 (63.70)	262 (62.38)	419 (64.56)	0.469
Age, M (Q_1_, Q_3_)	61.00 (49.00, 70.00)	56.00 (46.00, 66.00)	67.00 (56.00, 77.00)	<0.001
Disease type, *N* (%)				<0.001
TBI	413 (38.63)	96 (22.86)	317 (48.84)	
ICH	490 (45.84)	285 (67.86)	205 (31.59)	
SAH	166 (15.53)	39 (9.29)	127 (19.57)	
Surgical intervention, *N* (%)	426 (39.85)	202 (48.10)	224 (34.51)	<0.001
ICU admission, *N* (%)	805 (75.30)	388 (92.38)	417 (64.25)	<0.001
Length of ICU stay, M (Q_1_, Q_3_)	8.00 (3.00, 19.00)	5.00 (2.00, 13.00)	14.00 (7.00, 26.00)	<0.001
Days of hospitalization, M (Q_1_, Q_3_)	22.00 (14.00, 33.00)	23.00 (16.00, 33.00)	20.00 (11.00, 33.00)	<0.001
Onset to admit hours, M (Q_1_, Q_3_)	5.00 (3.00, 7.00)	5.00 (3.00, 8.00)	4.00 (3.00, 6.00)	<0.001
TBI	5.00 (4.00, 8.00)	6.00 (4.00, 9.00)	5.00 (4.00, 7.00)	
ICH	4.00 (3.00, 6.00)	4.00 (3.00, 8.00)	4.00 (3.00, 6.00)	
aSAH	4.00 (3.00, 6.00)	4.00 (3.00, 6.00)	4.00 (2.00, 6.00)	
GCS admit category, *N* (%)				<0.001
Mild (13–15)	625 (58.47)	499 (76.89)	126 (30.00)	
Moderate (9–12)	225 (21.05)	104 (16.02)	121 (28.81)	
Severe (3–8)	219 (20.49)	46 (7.09)	173 (41.19)	
Comorbidites				
Hypertension, *N* (%)	556 (52.01)	226 (53.81)	330 (50.85)	0.344
Diabetes, *N* (%)	173 (16.18)	79 (18.81)	94 (14.48)	0.061
Coronary heart disease (CHD), *N* (%)	136 (12.72)	65 (15.48)	71 (10.94)	0.030
Stroke/hemorrhage, *N* (%)	188 (17.59)	101 (24.05)	87 (13.41)	<0.001
Primary liver function marker				
AAR, M (Q_1_, Q_3_)	1.09 (0.78, 1.50)	1.01 (0.75, 1.35)	1.18 (0.85, 1.69)	<0.001

Data are presented as median (interquartile range [IQR]) for continuous variables and number (percentage) for categorical variables. *p*-values were calculated using the Mann–Whitney U test for continuous variables and the chi-square test (or Fisher’s exact test where appropriate) for categorical variables. Favorable outcome was defined as a modified Rankin Scale (mRS) score of 0–2; unfavorable outcome was defined as mRS 3–6.

AAR, aspartate aminotransferase-to-alanine aminotransferase ratio; aSAH, aneurysmal subarachnoid hemorrhage; CHD, coronary heart disease; GCS, Glasgow Coma Scale; ICH, intracerebral hemorrhage; ICU, intensive care unit; TBI, traumatic brain injury.

At hospital discharge, 649 patients (60.7%) achieved favorable functional outcomes, whereas 420 patients (39.3%) experienced unfavorable outcomes. Patients with unfavorable outcomes were significantly older (median 67 vs. 56 years, *p* < 0.001), had longer ICU stays (median 14 vs. 5 days, *p* < 0.001), and presented with more severe neurological impairment at admission (GCS 3–8: 41.2% vs. 7.1%, *p* < 0.001). Medication analysis revealed that specific interventions, including albumin supplementation and *β*-lactamase inhibitors, were more frequently administered to patients with unfavorable outcomes, likely reflecting greater illness severity and treatment intensity. Onset-to-admission time was incorporated into subsequent adjusted analyses to account for potential timing-related effects on systemic biomarkers (Supplementary Table S1).

### Liver function parameters and AAR distribution

**Figure 1:**
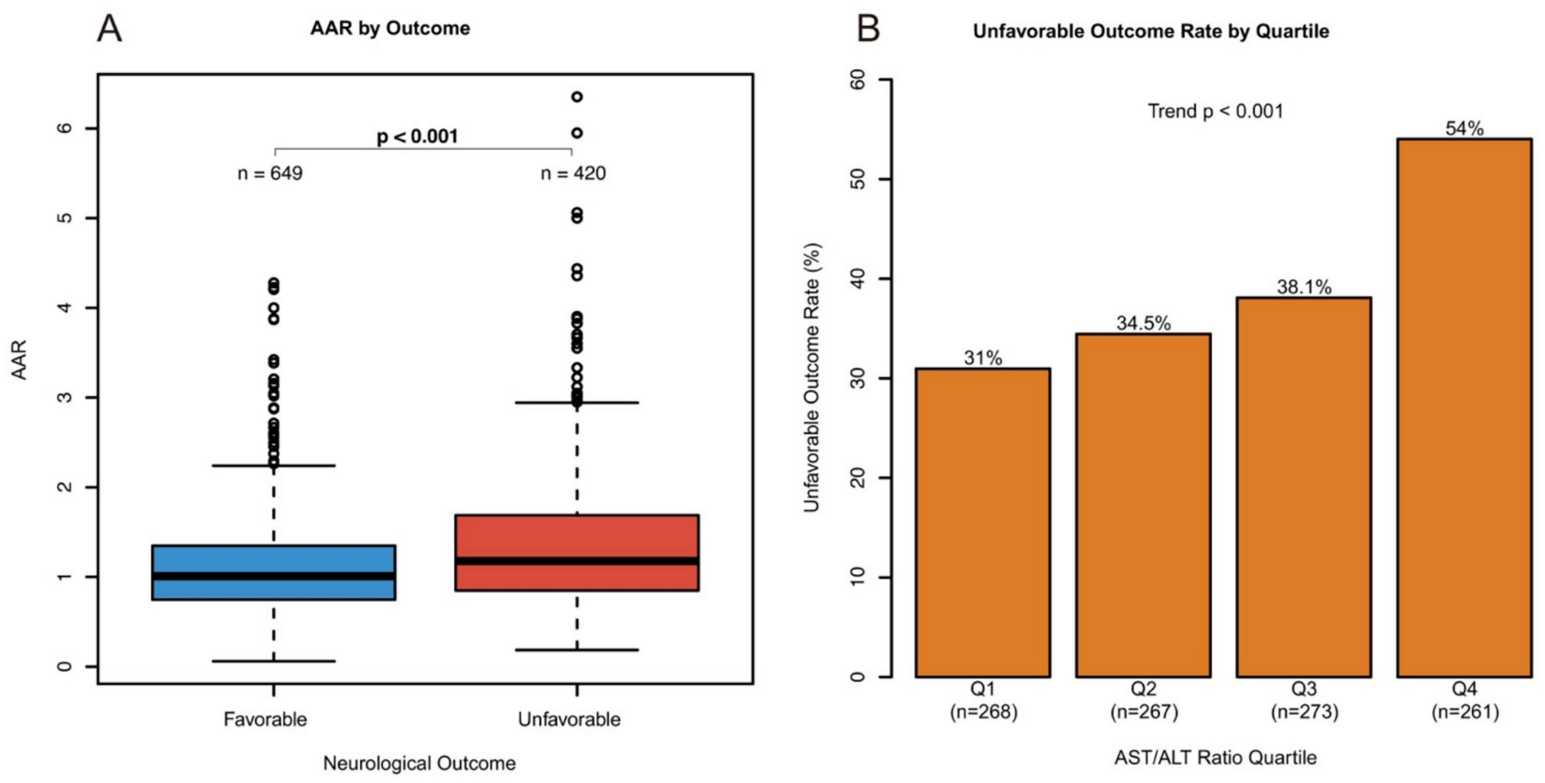
AAR distribution and dose–response relationship with neurological outcomes. **(A)** Shows the distribution of AAR values based on neurological outcome. Patients with unfavorable outcomes (mRS 3–6, *n* = 420) had significantly higher median AAR compared to those with favorable outcomes (mRS 0–2, *n* = 649): 1.18 (IQR: 0.85–1.69) versus 1.01 (IQR: 0.75–1.35), *p* < 0.001. Box plots display the median (central line), interquartile range (box), and individual data points (dots). **(B)** Shows a significant dose–response relationship between AAR quartiles and unfavorable outcome rates. The rate increased across quartiles: Q1 (31%), Q2 (34.5%), Q3 (38.1%), and Q4 (54%), with a significant linear trend (*p* < 0.001). This gradient supports AAR as a continuous predictor rather than a simple dichotomous marker.

Detailed laboratory analysis revealed a high prevalence of subclinical hepatic stress (Supplementary Table S2). Although 67.8% of the cohort maintained normal liver function according to established criteria, 32.2% exhibited some degree of acute liver injury (24.5% mild, 5.0% moderate, and 2.6% severe). The median AAR was significantly higher in patients with unfavorable outcomes compared to those with favorable outcomes (1.18 [IQR: 0.85–1.69] vs. 1.01 [0.75–1.35], *p* < 0.001). This elevation in AAR was primarily driven by a disproportionate increase in AST levels rather than ALT. Notably, a significant dose–response relationship was observed, with the rate of unfavorable outcomes increasing linearly across AAR quartiles: Q1 (31.0%), Q2 (34.5%), Q3 (38.1%), and Q4 (54.0%) (P for trend < 0.001) (Figure 1).

### Multivariable predictors in the overall cohort

**Figure 2:**
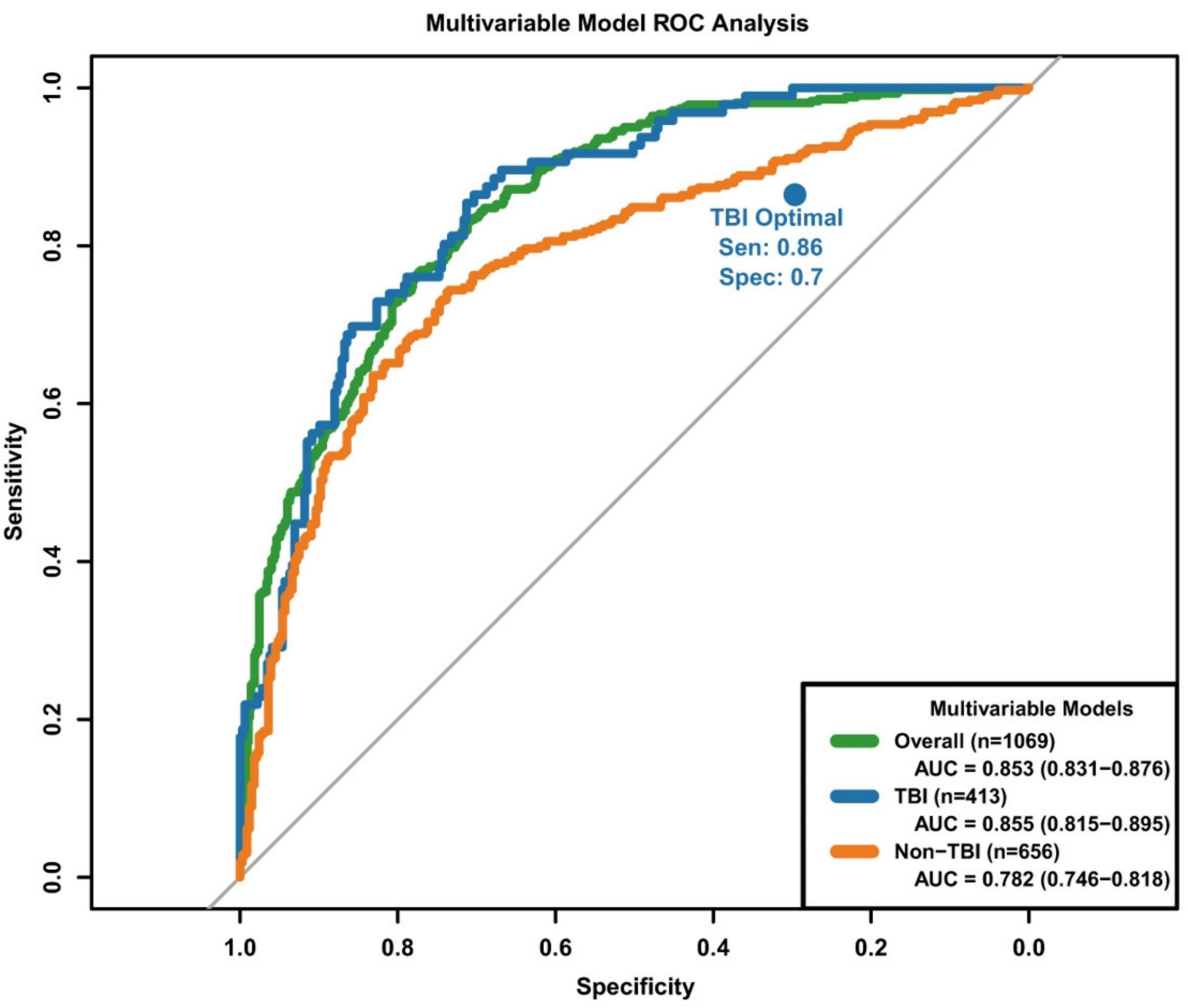
Receiver operating characteristic curves for multivariable models predicting unfavorable neurological outcomes. The ROC curves illustrate the discriminative ability of multivariable logistic regression models that include AAR and other clinical variables. The overall model (green line) achieved an AUC of 0.853 (95% CI, 0.831–0.876), with an optimal sensitivity of 86% and specificity of 71%. Disease-specific models showed different performance: the TBI subgroup (blue line) had an AUC of 0.862 with sensitivity 86% and specificity 69%; the non-TBI subgroup (orange line) had an AUC of 0.782 (95% CI, 0.748–0.816). The higher performance in TBI patients indicates that AAR has greater prognostic value in this group. Models were adjusted for age, disease type, ICU length of stay, surgical intervention, LDH, and creatinine levels. The diagonal line represents chance performance (AUC = 0.50).

To isolate the independent prognostic value of AAR, a multivariable logistic regression model was constructed to adjust for demographics, disease type, clinical severity, and other laboratory parameters (Table 2). The model demonstrated good discrimination, with an area under the receiver operating characteristic (ROC) curve (AUC) of 0.853 (95% CI: 0.831–0.876), corresponding to a sensitivity of 86% and a specificity of 71% at the optimal threshold (Figure 2). In the overall cohort, higher AAR remained independently associated with unfavorable outcomes (adjusted OR 1.34 per unit increase, 95% CI: 1.07–1.66, *p* = 0.002). Other independent predictors included older age (adjusted OR 1.06, *p* < 0.001), prolonged ICU stay (adjusted OR 1.02, *p* < 0.001), and the diagnosis of ICH (adjusted OR 4.62 compared to TBI, *p* < 0.001).

**Table 2:** Predictors of unfavorable neurological outcome: logistic regression analysis.

Variable	Adjusted OR (95% CI)	*p*-value
Demographics		
Age (per year)	1.06 (1.04–1.07)	<0.001
Male	—	—
Disease type		
TBI	1.00 (reference)	—
ICH	4.62 (3.19–6.68)	<0.001
aSAH	0.89 (0.53–1.49)	0.655
Clinical severity		
ICU length of stay (Per day)	1.02 (1.01–1.04)	<0.001
Surgical intervention	2.02 (1.44–2.84)	<0.001
Laboratory parameters		
AAR (per unit)	1.41 (1.13–1.76)	0.002
LDH (per 10 u/l)	1.01 (1.01–1.01)	<0.001
Albumin/total protein ratio	0.07 (0.00–1.00)	0.050
Creatinine (per 10 μmoL/L)	1.01 (1.01–1.01)	0.006

The multivariable model was developed using variables with *p* < 0.10 in the univariate analysis and those biologically plausible. Model discrimination was assessed using the area under the ROC curve (AUC = 0.853, 95% CI: 0.831–0.876), and calibration was assessed using the Hosmer–Lemeshow test (*p* = 0.394). Nagelkerke *R*^2^ was 0.352.

AAR, aspartate aminotransferase-to-alanine aminotransferase ratio; aSAH, aneurysmal subarachnoid hemorrhage; CI, confidence interval; ICH, intracerebral hemorrhage; ICU, intensive care unit; LDH, lactate dehydrogenase; OR, odds ratio; TBI, traumatic brain injury.

### Disease-specific subgroup analysis and interaction testing

**Figure 3:**
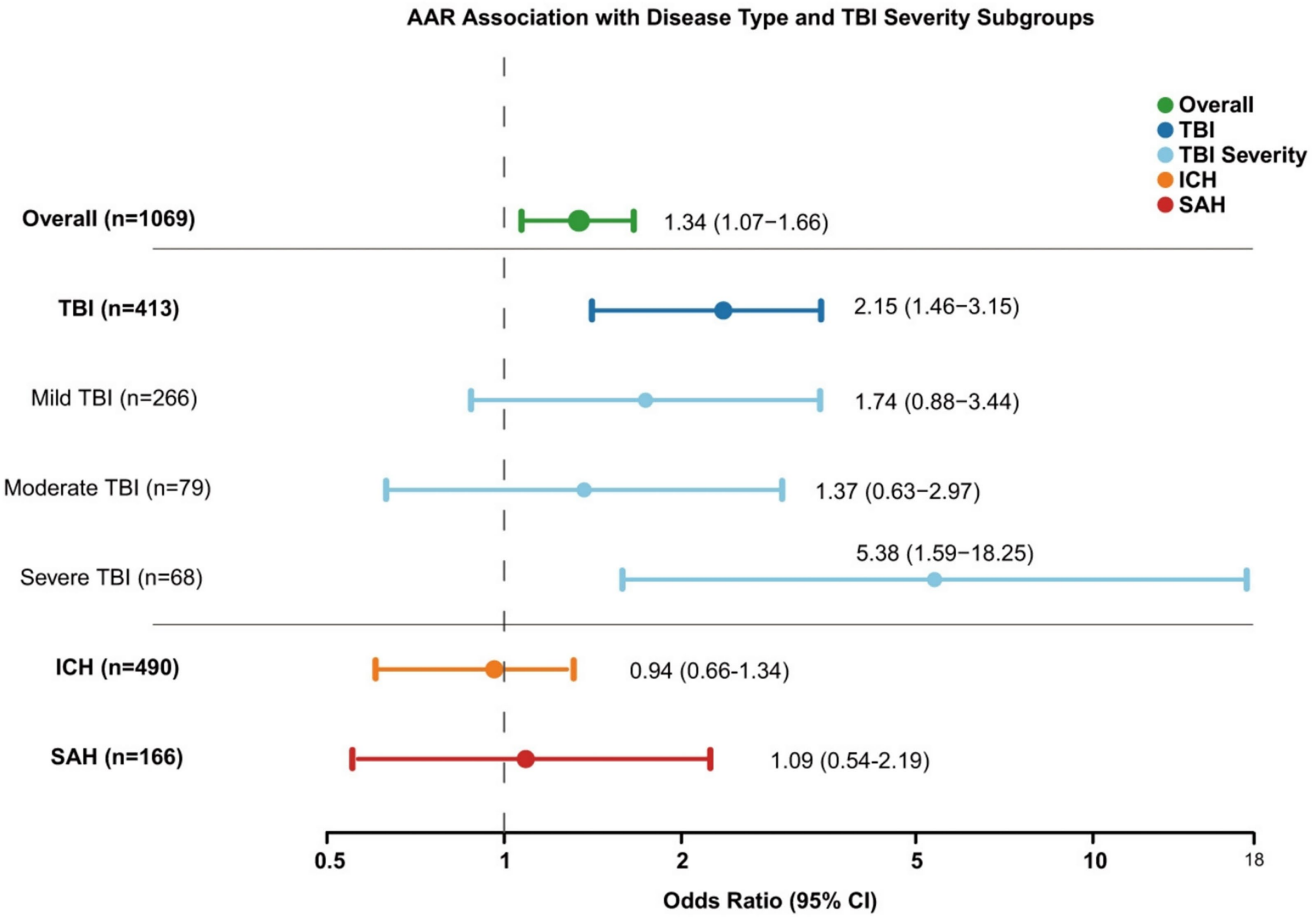
Disease-specific association of AAR with neurological outcomes and TBI severity subgroup analysis. Forest plot showing adjusted odds ratios (OR) with 95% confidence intervals for the association between AAR (per unit increase) and unfavorable neurological outcomes across different disease types and TBI severity subgroups. The overall cohort (*n* = 1,069) demonstrated a modest association (OR 1.34, 95% CI: 1.07–1.66). However, subgroup analysis revealed significant heterogeneity: TBI patients (*n* = 413) exhibited a strong association (OR 2.15, 95% CI: 1.46–3.15), with the strongest effect in severe TBI (GCS 3–8, *n* = 68): OR 5.38 (95% CI: 1.59–18.25). Mild (*n* = 286) and moderate TBI (*n* = 79) showed intermediate associations. In contrast, ICH (*n* = 490, OR 0.94, 95% CI: 0.66–1.34) and aSAH (*n* = 166, OR 1.09, 95% CI: 0.54–2.19) showed no significant associations. The vertical dashed line at OR = 1 marks the null hypothesis. Formal interaction testing confirmed significant effect modification by disease type (*p* = 0.001), indicating notably different pathophysiological mechanisms behind AAR’s prognostic value across different acute brain injury subtypes.

Given the distinct pathophysiological mechanisms among ABI subtypes, we conducted a pre-specified subgroup analysis (Table 3 and Figure 3). Striking heterogeneity was observed in the prognostic value of AAR. In the TBI subgroup, AAR emerged as a potent independent predictor of unfavorable outcomes (adjusted OR 2.15, 95% CI: 1.46–3.15, *p* < 0.001). Conversely, AAR showed no significant association with outcomes in patients with ICH (adjusted OR 0.94, 95% CI: 0.66–1.34, *p* = 0.746) or aSAH (adjusted OR 1.09, 95% CI: 0.54–2.19, *p* = 0.810). Formal interaction testing confirmed a significant effect modification by disease type (P for interaction = 0.001), indicating that the predictive utility of AAR is highly specific to the etiology of brain injury. Consistent with this pattern, disease-specific ROC analysis showed markedly superior discrimination in the TBI subgroup (AUC 0.862, sensitivity 86%, specificity 69%) compared with the non-TBI cohort (AUC 0.782, 95% CI: 0.748–0.816) (Figure 2).

**Table 3:** Disease-specific association of AAR with unfavorable outcome and interaction analysis.

Subgroup	No. of patients	Median AAR [IQR] (favorable vs. unfavorable)	Adjusted OR (95% CI)*	*p*-value	P for interaction
Overall cohort	1,069	1.01 [0.75–1.35] vs. 1.18 [0.85–1.69]	1.34 (1.07–1.66)	0.010	—
By disease type					0.001
Traumatic brain injury (TBI)	413	0.97 [0.71–1.27] vs. 1.50 [0.95–1.98]	2.15 (1.46–3.15)	<0.001	Reference
Intracerebral hemorrhage (ICH)	490	1.06 [0.78–1.38] vs. 1.13 [0.81–1.55]	0.94 (0.66–1.34)	0.746	<0.001
Aneurysmal SAH (aSAH)	166	1.11 [0.85–1.42] vs. 1.24 [1.03–1.91]	1.09 (0.54–2.19)	0.810	0.069
By TBI severity					
Mild TBI (GCS 13–15)	286	—	1.74 (0.88–3.44)	0.112	—
Moderate TBI (GCS 9–12)	79	—	1.37 (0.63–2.97)	0.428	—
Severe TBI (GCS 3–8)	68	—	5.38 (1.59–18.25)	0.007	—

*Adjusted for age, ICU length of stay, surgical intervention, LDH, albumin-to-total protein ratio, alkaline phosphatase, creatinine, albumin, and urea.

The *p*-value for interaction represents the statistical significance of the difference in the prognostic effect of AAR between the specific disease subgroup and the reference group (TBI). The overall interaction test for disease type was significant (*χ*^2^ = 13.31, *p* = 0.001).

Subgroup analysis within the TBI cohort was performed to assess the impact of injury severity (GCS score) on the prognostic value of AAR.

AAR, aspartate aminotransferase-to-alanine aminotransferase ratio; aSAH, aneurysmal subarachnoid hemorrhage; CI, confidence interval; GCS, Glasgow Coma Scale; ICH, intracerebral hemorrhage; IQR, interquartile range; OR, odds ratio; TBI, traumatic brain injury.

### Impact of TBI severity

Further analysis within the TBI cohort revealed that the prognostic strength of AAR escalated with injury severity (Table 3). The association was non-significant in mild-to-moderate TBI but became exceptionally strong in the severe TBI subgroup (GCS 3–8), where each unit increase in AAR was associated with a more than 5-fold increase in the odds of an unfavorable outcome (adjusted OR 5.38, 95% CI: 1.59–18.25, *p* = 0.007). This pattern suggests that AAR’s prognostic value is maximized under conditions of severe neurotrauma.

### Sensitivity analyses accounting for biomarker timing

To assess the robustness of our findings, we conducted two pre-specified sensitivity analyses (Supplementary Table S3). First, additional adjustment for onset-to-admission time did not materially alter the disease-specific pattern: AAR remained an independent predictor of unfavorable outcome in the TBI subgroup (adjusted OR 2.21, 95% CI 1.35–3.63, *p* = 0.002), and the interaction by disease type remained significant (P for interaction = 0.016). Second, when the first available AAR measurement replaced the median in-hospital AAR, the TBI association was directionally consistent but attenuated and no longer statistically significant (adjusted OR 1.60, 95% CI 0.89–2.86, *p* = 0.113; P for interaction = 0.170), suggesting that the prognostic signal of AAR in TBI is more robustly captured by the overall in-hospital transaminase profile than by a single early measurement.

## Discussion

The present study demonstrates marked disease-specific heterogeneity in the prognostic value of AAR across major forms of acute brain injury. Although higher AAR was independently associated with unfavorable neurological outcome in the overall cohort, this signal was driven predominantly by the TBI subgroup. In patients with TBI, elevated AAR remained a robust independent predictor of poor outcome, and its effect size increased substantially with greater injury severity (adjusted OR 5.38 in severe TBI). In contrast, no significant association was observed in patients with ICH or aSAH. Formal interaction testing further confirmed that the prognostic association between AAR and outcome differed significantly by disease type (P for interaction = 0.001). Taken together, these findings suggest that AAR should not be regarded as a universally transferable biomarker across neurocritical conditions, but rather as a marker whose clinical relevance may depend strongly on the underlying injury context.

Our findings align with emerging evidence indicating a possible link between liver function markers and neurological outcomes. Tsai et al. previously reported that changes in the De Ritis ratio (ΔDRR) could help stratify mortality risk in patients with moderate-to-severe TBI, with a ΔDRR of 0.7 serving as a cutoff point for mortality risks (17), suggesting that the pattern of liver enzyme changes over time might be more informative than their absolute values. A large propensity score-matched analysis by Lin et al. corroborated these findings in a cohort of 4,415 patients with moderate-to-severe TBI (head/neck AIS ≥ 3), demonstrating that an initial AAR > 1.64 was independently associated with significantly higher in-hospital mortality compared with the reference group (17.6% vs. 8.2%, OR = 1.33, 95% CI, 1.01–1.77, *p* = 0.046) (18). Importantly, the use of propensity score matching in that study mitigates confounding by baseline severity, lending additional causal credibility to the AAR–TBI mortality relationship. Previous research has shown that AAR is not only associated with adverse outcomes in hemorrhagic stroke but also linked to poor prognosis in ischemic stroke. Gao et al. demonstrated that an AAR > 1.53 at admission was connected to worse 3-month outcomes in acute ischemic stroke patients (OR 2.15, 95% CI: 1.14–4.05) (9). Similarly, Xu et al. found that AAR was related to all-cause mortality and poor functional outcomes in stroke patients (19), though their study did not specifically focus on TBI. The consistency of our results with these previous studies suggests that the specific association of AAR with TBI outcomes may reflect the unique pathophysiological processes following TBI.

Unlike other hemorrhagic and ischemic strokes, TBI triggers a complex cascade involving glutamate metabolism disorder (20), sustained catecholamine release (21), and systemic inflammatory activation (22) that seem to create conditions particularly conducive to hepatic dysfunction. Together, these pathophysiological mechanisms support the concept that TBI induces a distinctive multi-organ metabolic crisis, of which elevated AAR may represent the hepatic manifestation. In the acute phase of TBI, a large amount of glutamate enters the extracellular space due to cell membrane damage and abnormal release of synaptic vesicles, and the buildup of extracellular glutamate leads to excitotoxicity (20). This may also explain the short-term rapid increase of AST after TBI. Gao et al.’s glutamate metabolism hypothesis suggests that elevated transaminases facilitate glutamate clearance from the blood, creating a brain-to-blood concentration gradient that accelerates glutamate efflux from injured brain tissue (9). The sustained elevation of AAR we observed in TBI patients might reflect the ongoing demand for peripheral glutamate clearance—a protective mechanism that paradoxically signals greater injury severity. TBI also causes a prolonged catecholamine storm linked to functional outcomes and leading to hepatic metabolic stress (23, 24), evidenced by elevated AAR from mitochondrial AST release (25). At the same time, catecholamines shift immune responses from Th1 to Th2 pathways via β2-adrenergic receptors, suppressing pro-inflammatory and promoting anti-inflammatory cytokines (26).

The higher elevation of AST relative to ALT in our TBI cohort suggests mitochondrial dysfunction as a key mechanism. The underlying reason for AAR changes mainly involves the differing release of mitochondrial versus cytoplasmic enzymes. AST is found in both cytoplasmic and mitochondrial forms within liver cells, while ALT is mainly cytoplasmic (25). Under normal conditions, circulating AST levels primarily come from the cytoplasmic portion, but severe cellular injury causes the release of both cytoplasmic and mitochondrial AST, increasing the AAR (27). This increase in ratio indicates the degree of mitochondrial damage and energy failure in cells. TBI triggers a unique, sustained hepatic dysfunction through a multi-pathway cascade. This involves the acute phase response, cytokine storm, and bile acid transport defects, creating a distinct neurometabolic signature not observed in hemorrhagic conditions (28, 29). TBI-specific disruption of bile acid homeostasis—through loss of hypothalamic ASBT-expressing neurons and decreased TGR5 expression—establishes a direct brain-liver metabolic regulatory link (28).

The lack of AAR prognostic value in ICH and SAH deserves more careful analysis. These conditions involve different timing patterns of brain injury—ICH causes immediate mass effect and local tissue damage (30), while SAH leads to acute vasospasm and delayed ischemia (31). Additionally, the connection between pre-existing liver problems and hemorrhagic stroke might interfere with the prognostic usefulness of AAR in these cases. Research indicates that liver disease itself increases the risk of ICH through mechanisms that depend on or are independent of coagulopathy (32, 33). In our group, patients with hemorrhagic strokes might have had underlying liver issues that hid any injury-related AAR changes. It is also important to acknowledge that TBI and hemorrhagic stroke are not mutually exclusive entities. In our cohort, traumatic subarachnoid hemorrhage was classified under TBI rather than aSAH, which may have contributed to the stronger AAR signal observed in that group. Furthermore, ICH itself is mechanistically heterogeneous. Hypertensive small vessel disease leads to deep hemorrhages (basal ganglia, thalamus, brainstem) through lipohyalinosis-driven rupture, whereas cerebral amyloid angiopathy (CAA)-related hemorrhage involves cortical and leptomeningeal *β*-amyloid deposition, manifesting as lobar bleeds with a distinct neuroinflammatory milieu and a tendency for recurrence (34, 35). Hypertensive ICH primarily causes acute focal mass effect with relatively contained systemic metabolic consequences, whereas CAA-related hemorrhage may trigger broader vascular inflammatory responses (36, 37). This etiological heterogeneity within our ICH cohort—pooling hypertensive and CAA-related cases without stratification—may have further diluted any subtype-specific association between AAR and systemic metabolic stress. Future investigations stratifying ICH by etiology will be essential to determine whether hypertensive and CAA-related hemorrhage exhibit differential AAR profiles.

Despite the disease-specific heterogeneity we identified, certain shared pathophysiological substrates warrant consideration. Acute neuroinflammation, blood–brain barrier disruption, and systemic oxidative stress are common to TBI, ICH, and aSAH, albeit with differing magnitudes and temporal dynamics. A key lesson from our multi-etiology cohort is precisely that comparing across disease types within a single study reveals what single-disease investigations cannot: the liver-brain axis is engaged differently depending on injury mechanism, not uniformly activated by all forms of acute brain injury. The null finding in ICH and aSAH is not a failure of the biomarker but a scientifically informative result that delineates the boundaries of AAR utility. Moreover, TBI itself confers a substantially elevated long-term risk of subsequent stroke—a recent meta-analysis demonstrated a pooled hazard ratio of 1.59 (95% CI, 1.37–1.85) for any stroke following TBI, with a particularly striking association for hemorrhagic stroke (HR = 4.68, 95% CI: 2.93–7.49) (38)—suggesting that the systemic vascular and metabolic consequences of TBI extend well beyond the acute hospitalization period and may eventually converge with hemorrhagic stroke pathophysiology. This temporal overlap further reinforces the value of multi-etiology cohort designs in capturing the continuum of brain injury biology.

The disease-specific associations we observed could have therapeutic implications. If AAR elevation in TBI indicates ongoing glutamate toxicity and mitochondrial dysfunction, targeted interventions addressing these mechanisms might improve outcomes (39, 40). N-acetylcysteine, which enhances glutathione synthesis and has mitochondrial protective effects, is one such approach that has shown promise in acute liver failure (41) and may warrant investigation in TBI (42, 43). From an anticoagulation and antiplatelet perspective, the divergent AAR profiles across injury types carry direct clinical relevance. TBI patients face the well-recognized dilemma of balancing venous thromboembolism prophylaxis against ongoing intracranial hemorrhagic risk, with prior evidence indicating that delayed pharmacologic prophylaxis is associated with higher VTE rates while earlier initiation may raise concern for hematoma progression—leaving the optimal timing unresolved (44). Given that AST predominantly reflects mitochondrial enzyme release and that transaminase elevations have been proposed as sensitive indicators of systemic metabolic stress in critically ill patients (27), serial AAR measurement in TBI may warrant formal evaluation as an accessible complementary signal to existing clinical and imaging criteria when weighing prophylaxis timing. In contrast, contemporary ICH guidelines prioritize immediate anticoagulation reversal as the primary hemostatic intervention (45), and our null finding in ICH suggests that AAR is unlikely to provide meaningful incremental prognostic information beyond established neuroimaging and coagulation-based risk stratification frameworks in this setting.

Regarding intracranial pressure (ICP) monitoring, current Brain Trauma Foundation guidelines recommend invasive ICP monitoring in salvageable severe TBI patients with GCS 3–8 and abnormal admission CT findings (46). The pronounced escalation of AAR prognostic strength with TBI severity observed in our cohort (adjusted OR 5.38 in GCS 3–8 patients) is biologically consistent with the established framework that severe TBI triggers a sympathetic catecholamine surge (21) and mitochondrial bioenergetic failure (42), which may concurrently drive intracranial hypertension and peripheral metabolic derangement through shared neuroendocrine pathways. However, whether dynamic AAR trajectories correlate with ICP trends has not been directly examined in humans. Our findings should therefore be regarded strictly as hypothesis-generating. Prospective studies integrating serial transaminase measurements with continuous ICP recordings will be required before AAR could be considered as a complementary, non-invasive signal to support neurocritical care decision-making.

Our findings should also be interpreted alongside the rapidly evolving landscape of molecular biomarkers in hemorrhagic brain injury. Non-coding RNAs—including miRNAs, lncRNAs, and circRNAs—have been increasingly recognized as key regulators of neuroinflammatory pathways in hemorrhagic stroke, such as NF-κB, PI3K/AKT, and NLRP3 inflammasome signaling (47). NLRP3 activation in perihematomal tissue (48) and proinflammatory miRNAs such as miR-155 (49) have been specifically implicated in ICH-related injury. Importantly, AAR and ncRNA biomarkers likely capture complementary, rather than competing, dimensions of brain injury biology: AAR indexes systemic and hepatic metabolic stress through the liver-brain axis, whereas ncRNA signatures reflect local CNS molecular regulation. This distinction offers one plausible framework for our disease-specific pattern—TBI, with pronounced systemic metabolic consequences, showed a strong AAR signal, whereas ICH and aSAH, whose pathology is more regionally confined, did not. The null findings in ICH and aSAH thus argue less for a limitation of AAR than for the development of multi-modal prognostic frameworks integrating systemic and CNS-targeted biomarkers across etiologies.

### Limitations

This study has several limitations. First, its retrospective single-center design limits causal inference and raises the possibility of residual confounding despite multivariable adjustment. Second, although onset-to-admission time was available for all patients and was included in sensitivity analyses, the precise timing of each laboratory test relative to injury onset, admission, and clinical interventions may still have varied across patients. Third, the primary AAR exposure was based on median in-hospital AST and ALT values, which may capture the overall biochemical burden during hospitalization rather than a purely baseline biomarker. To address this issue, we performed sensitivity analyses using the first available AAR measurement, but prospective studies with standardized sampling windows are still needed. Fourth, disease categories used for analysis inevitably simplified clinically heterogeneous entities. In particular, hemorrhagic lesions differ in etiology and mechanism, and traumatic and hemorrhagic phenotypes may overlap in real-world practice. Finally, the biological mechanisms underlying the stronger association of AAR with TBI outcomes remain inferential and were not directly measured. External validation in independent cohorts and integration with dynamic biomarker, imaging, and molecular data will be necessary before AAR can be incorporated into broader prognostic frameworks for neurocritical care.

## Conclusion

In conclusion, AAR is not a generic marker of poor prognosis in neurosurgery but a specific predictor of unfavorable outcomes in TBI, particularly in severe cases. Its lack of prognostic value in ICH and aSAH underscores the heterogeneity of liver-brain interactions across different neurocritical conditions. Monitoring AAR in TBI patients offers a low-cost, accessible window into systemic mitochondrial function, potentially guiding more aggressive metabolic support.

### Sample size justification

No formal *a priori* power calculation was performed due to the retrospective study design. However, the sample size was sufficient for multivariable logistic regression modeling based on the “Events Per Variable” (EPV) rule. With 420 unfavorable outcome events and approximately 10 predictors in the final model, the EPV ratio (>40:1) well exceeded the recommended minimum of 10:1, ensuring statistical stability and minimizing overfitting.

### Blinding

Outcome assessment (mRS at discharge) was extracted by investigators blinded to the specific liver function parameters and AAR values to prevent assessment bias.

### Missing data

Variables with >20% missing data were excluded. For variables with <10% missing data, missingness was assumed to be random, and complete case analysis was performed.

### Statistical analysis

The primary interaction analysis was pre-specified based on the biological hypothesis. All analyses were conducted using R software (version 4.3.2).

## Data Availability

The original contributions presented in the study are included in the article/Supplementary material, further inquiries can be directed to the corresponding authors.
