# Supplementary material for: Distinct prognostic value of aspartate-to-alanine aminotransferase ratio (AAR) in traumatic brain injury versus hemorrhagic stroke: a cohort study of 1,069 patients
Source: Front Neurol. 2026 Jun 4;17:1796615. doi: 10.3389/fneur.2026.1796615 (PMC13275345; doi:10.3389/fneur.2026.1796615)
Supplement: Supplementary file 1 [file Table_1.DOCX]

**Supplementary Table 1. Medications Administered During Hospitalization Stratified by Neurological Outcome**

| Supplementary Table 1. Medications Administered During Hospitalization Stratified by Neurological Outcome | | | | |
| --- | --- | --- | --- | --- |
| Variables | Total  (n = 1,069) | Favorable outcome (n=649) | Unfavorable outcome (n=420) | P-Value |
| Hemostatic, N(%) | 1,019 (95.32) | 392 (93.33) | 627 (96.61) | 0.013 |
| Acid suppressants, N(%) | 1,058 (98.97) | 414 (98.57) | 644 (99.23) | 0.465 |
| Expectorants, N(%) | 952 (89.06) | 383 (91.19) | 569 (87.67) | 0.072 |
| Osmotic agents, N(%) | 950 (88.87) | 372 (88.57) | 578 (89.06) | 0.804 |
| Valproate, N(%) | 456 (42.66) | 171 (40.71) | 285 (43.91) | 0.302 |
| Other antiepileptics, N(%) | 208 (19.46) | 67 (15.95) | 141 (21.73) | 0.020 |
| Analgesics and sedatives, N(%) | 684 (63.99) | 262 (62.38) | 422 (65.02) | 0.379 |
| Hepatoprotective agents, N(%) | 238 (22.26) | 98 (23.33) | 140 (21.57) | 0.499 |
| Albumin, N(%) | 290 (27.13) | 147 (35.00) | 143 (22.03) | <.001 |
| Cephalosporins, N(%) | 686 (64.17) | 272 (64.76) | 414 (63.79) | 0.746 |
| β-lactamase inhibitors, N(%) | 476 (44.53) | 223 (53.10) | 253 (38.98) | <.001 |
| Carbapenems, N(%) | 271 (25.35) | 127 (30.24) | 144 (22.19) | 0.003 |
| Vancomycin, N(%) | 130 (12.16) | 40 (9.52) | 90 (13.87) | 0.034 |
| Tigecycline, N(%) | 71 (6.64) | 31 (7.38) | 40 (6.16) | 0.435 |
| Linezolid, N(%) | 80 (7.48) | 27 (6.43) | 53 (8.17) | 0.292 |

**Notes:**Data are presented as number (percentage). P-values were calculated using the chi-square test or Fisher's exact test. "Osmotic agents" include mannitol, glycerol fructose, and hypertonic saline. "β-lactamase inhibitors" refer to antibiotics containing beta-lactamase inhibitors (e.g., sulbactam or tazobactam combinations). The high usage of acid suppressants reflects routine protocol for stress ulcer prophylaxis.

**Supplementary Table 2. Detailed Liver Function and Laboratory Parameters by Neurological Outcome**

| Supplementary Table 2. Detailed Liver Function and Laboratory Parameters by Neurological Outcome | | | | |
| --- | --- | --- | --- | --- |
| Variables | Total  (n = 1,069) | Favorable outcome (n=649) | Unfavorable outcome (n=420) | P-Value |
| **Primary liver function marker** |  |  |  |  |
| AAR, M (Q₁, Q₃) | *1.09 (0.78, 1.50)* | *1.01 (0.75, 1.35)* | *1.18 (0.85, 1.69)* | *<.001* |
| **Individual liver enzymes** |  |  |  |  |
| ALT (U/L), M (Q₁, Q₃) | *30.50 (21.00, 46.50)* | *29.00 (20.00, 43.00)* | *32.75 (22.50, 51.00)* | *<.001* |
| AST (U/L), M (Q₁, Q₃) | *32.00 (23.00, 47.00)* | *28.50 (21.50, 40.50)* | *38.75 (26.00, 59.62)* | *<.001* |
| ALKP (U/L), M (Q₁, Q₃) | *78.50 (63.00, 100.00)* | *75.00 (60.00, 94.00)* | *85.00 (68.00, 109.00)* | *<.001* |
| GGT (U/L), M (Q₁, Q₃) | *32.00 (20.00, 69.00)* | *29.50 (18.50, 56.50)* | *37.00 (22.00, 78.50)* | *<.001* |
| **Protein synthesis markers** |  |  |  |  |
| Albumin (g/L), M (Q₁, Q₃) | *35.50 (32.00, 39.00)* | *37.00 (34.00, 40.00)* | *33.00 (30.00, 36.50)* | *<.001* |
| Total protein (g/L), M (Q₁, Q₃) | *66.00 (62.00, 71.00)* | *67.00 (63.00, 71.50)* | *64.00 (60.00, 68.62)* | *<.001* |
| Albumin/Total protein ratio, M (Q₁, Q₃) | *0.54 (0.50, 0.57)* | *0.55 (0.52, 0.58)* | *0.52 (0.49, 0.54)* | *<.001* |
| Globulin (g/L), M (Q₁, Q₃) | *31.00 (28.00, 34.00)* | *31.00 (28.00, 33.50)* | *31.00 (28.88, 34.00)* | *0.097* |
| **Other markers** |  |  |  |  |
| Creatinine (µmol/L), M (Q₁, Q₃) | *58.00 (47.50, 69.50)* | *57.00 (46.00, 66.50)* | *60.00 (49.00, 79.00)* | *<.001* |
| Urea (mmol/L), M (Q₁, Q₃) | *6.00 (4.75, 8.00)* | *5.45 (4.40, 6.60)* | *7.60 (5.85, 10.30)* | *<.001* |
| Uric acid (µmol/L), M (Q₁, Q₃) | *185.00 (131.50, 260.00)* | *192.00 (138.00, 261.00)* | *169.75 (119.00, 256.25)* | *0.012* |
| LDH (U/L), M (Q₁, Q₃) | *205.50 (172.50, 251.50)* | *195.00 (165.00, 233.00)* | *227.00 (190.97, 284.62)* | *<.001* |
| Total Bilirubin (µmol/L), Median (IQR) | *11.50 (8.60, 15.85)* | *11.70 (8.80, 16.05)* | *10.90 (8.29, 15.31)* | *0.052* |
| **Liver injury classification** |  |  |  | *0.002* |
| Normal | 725 (67.82) | 257 (61.19) | 468 (72.11) |  |
| Mild injury | 262 (24.51) | 123 (29.29) | 139 (21.42) |  |
| Moderate injury | 54 (5.05) | 25 (5.95) | 29 (4.47) |  |
| Severe injury | 28 (2.62) | 15 (3.57) | 13 (2.00) |  |
| **R ratio category, n(%)** |  |  |  | *0.796* |
| Hepatocellular(r≥5) | *49 (4.58)* | *17 (4.05)* | *32 (4.93)* |  |
| Mixed (2<r<5) | *246 (23.01)* | *97 (23.10)* | *149 (22.96)* |  |
| Cholestatic (r≤2) | *774 (72.40)* | *306 (72.86)* | *468 (72.11)* |  |
|  | | | | |

**Notes:**Data are presented as median (interquartile range [IQR]) for continuous variables and number (percentage) for categorical variables. P-values were calculated using the Mann-Whitney U test or chi-square test. Liver injury severity was classified according to established criteria: Mild injury (ALT 2–5 × ULN); Moderate injury (ALT 5–15 × ULN); Severe injury (ALT ≥10 × ULN with INR ≥2.0 and total bilirubin ≥3.0 mg/dL). R ratio = (ALT/ULN) / (ALKP/ULN) was used to classify the pattern of injury as hepatocellular (R ≥5), mixed (2 < R < 5), or cholestatic (R ≤2). **Abbreviations:** AAR, aspartate aminotransferase-to-alanine aminotransferase ratio; ALKP, alkaline phosphatase; ALT, alanine aminotransferase; AST, aspartate aminotransferase; GGT, gamma-glutamyl transferase; LDH, lactate dehydrogenase; ULN, upper limit of normal.

**Supplementary Table 3. Sensitivity Analyses: Association of AAR with Unfavorable Neurological Outcome Across Disease Subgroups**

| Supplementary Table 3. Sensitivity Analyses: Association of AAR with Unfavorable Neurological Outcome Across Disease Subgroups | | | | |
| --- | --- | --- | --- | --- |
| Model | Subgroup (n) | Adjusted OR (95% CI) | P value | P for interaction |
| Primary model  (median in-hospital AAR) | TBI (n=413) | 2.15 (1.46–3.15) | <0.001 | **0.001** |
|  | ICH (n=490) | 0.94 (0.66–1.34) | 0.746 |  |
|  | aSAH (n=166) | 1.09 (0.54–2.19) | 0.810 |  |
| Sensitivity Model 1  (additionally adjusted for onset-to-admission time) | TBI (n=413) | 2.21 (1.35–3.63) | 0.002 | **0.016** |
|  | ICH (n=490) | 1.01 (0.71–1.42) | 0.974 |  |
|  | aSAH (n=166) | 1.41 (0.77–2.58) | 0.270 |  |
| Sensitivity Model 2  (first available AAR instead of median) | TBI (n=413) | 1.60 (0.89–2.86) | 0.113 | 0.170 |
|  | ICH (n=490) | 1.04 (0.76–1.42) | 0.821 |  |
|  | aSAH (n=166) | 0.75 (0.36–1.56) | 0.444 |  |

**Notes:** Data are presented as adjusted odds ratios (OR) with 95% confidence intervals (CI) from multivariable logistic regression models predicting unfavorable neurological outcome (mRS 3–6) at hospital discharge. AAR was entered as a continuous variable, and ORs represent the change in odds of an unfavorable outcome per one-unit increase in AAR. In the primary model, AAR was defined as the ratio of the median in-hospital AST to the median in-hospital ALT; all models were adjusted for age, length of ICU stay, surgical intervention, serum lactate dehydrogenase, and serum creatinine. Sensitivity Model 1 additionally adjusted for onset-to-admission time (hours) to account for potential differences in the timing of biomarker sampling. Sensitivity Model 2 substituted the first available AAR measurement after admission for the median in-hospital AAR, with the same covariate adjustment as in the primary model. Subgroup analyses were conducted separately within each disease type (TBI, ICH, and aSAH). Formal interaction testing between AAR and disease type was performed using the likelihood ratio test comparing models with and without the AAR × disease-type interaction term. Bold values in the "P for interaction" column indicate statistically significant effect modification (P < 0.05). **Abbreviations:** AAR, aspartate aminotransferase–to–alanine aminotransferase ratio; ALT, alanine aminotransferase; AST, aspartate aminotransferase; aSAH, aneurysmal subarachnoid hemorrhage; CI, confidence interval; ICH, intracerebral hemorrhage; ICU, intensive care unit; mRS, modified Rankin Scale; OR, odds ratio; TBI, traumatic brain injury.
